# Comparative Evaluation of Concurrent Carboplatin-Paclitaxel Versus Cisplatin-Etoposide Chemoradiotherapy in Unresectable Non-small Cell Lung Cancer: A Prospective Randomized Study

**DOI:** 10.7759/cureus.116064

**Published:** 2026-09-10

**Authors:** Yamini Kumar, Bala Venkat Subramanian, G V Deepthi, Pranabandhu Das, Sreenivasa Rao Bonala

**Affiliations:** 1 Radiation Oncology, Sri Venkateswara Institute of Medical Sciences (SVIMS), Tirupati, IND

**Keywords:** carboplatin, cisplatin, concurrent chemoradiotherapy, etoposide, locally advanced nsclc, overall survival (os), paclitaxel, progression-free survival (pfs), radiation therapy, toxicity

## Abstract

Background: Concurrent chemoradiotherapy (CCRT) is the standard treatment for patients with unresectable non-small cell lung cancer (NSCLC). Cisplatin-etoposide (EP) and weekly carboplatin-paclitaxel (PC) are commonly used concurrent regimens, but comparative data on efficacy and toxicity remain limited. This study compared acute treatment-related toxicities and tumor response between these regimens, with six- and 12-month progression-free survival (PFS) and overall survival (OS) evaluated as pre-specified exploratory secondary outcomes.

Materials and methods: This prospective randomized study included 34 patients with histologically confirmed unresectable NSCLC treated between April 2021 and June 2022. Patients received either EP (n=16) or PC (n=18) concurrently with definitive radiotherapy (60 Gy in 30 fractions). Acute toxicities and three-month tumor response were evaluated. Kaplan-Meier PFS and OS were estimated separately through six and 12 months in 31 evaluable patients, with horizon-specific log-rank and Cox analyses.

Results: EP produced significantly more severe anemia, thrombocytopenia, and esophagitis, whereas pneumonitis was numerically more frequent with PC. The tumor-response distribution favored EP (p=0.029). Six-month PFS was 66.7% with EP and 56.3% with PC (p=0.499), and 12-month PFS was 40.0% and 25.0%, respectively (p=0.206). Six-month OS was 93.3% with EP and 81.3% with PC (p=0.317), and 12-month OS was 80.0% and 56.3%, respectively (p=0.144).

Conclusions: EP was associated with a more favorable tumor-response distribution and numerically better one-year PFS and OS than PC, but the survival differences were not statistically significant. These potential efficacy benefits were accompanied by greater hematological and esophageal toxicity.

## Introduction

Lung cancer is the leading cause of cancer-related mortality worldwide, with its incidence continuing to rise, particularly in developing countries [1]. Approximately 40%-50% of patients with non-small cell lung cancer (NSCLC) are diagnosed with unresectable locally advanced disease [2].

The management of locally advanced NSCLC requires a multidisciplinary approach that includes surgery, chemotherapy, radiotherapy, immunotherapy, targeted therapy, or a combination of these modalities, depending on disease stage and patient characteristics [3]. Concurrent chemoradiotherapy (CCRT) is the standard treatment for patients with unresectable NSCLC with good performance status [4] and has demonstrated a significant improvement in overall survival (OS) compared with sequential chemoradiotherapy [5]. However, these survival benefits are accompanied by an increased incidence of treatment-related toxicities, including esophagitis, neutropenia, and anemia [6]. The rationale for concurrent treatment is that radiotherapy provides effective locoregional tumor control, while chemotherapy enhances radiosensitivity and eradicates micrometastatic disease [7]. Reported five-year OS rates are approximately 25% with CCRT, 15% with sequential chemoradiotherapy, and less than 10% with radiotherapy alone [6]. Furthermore, CCRT has been shown to reduce both locoregional recurrence and distant metastasis compared with radiotherapy alone [8].

CCRT is considered the standard of care for unresectable NSCLC [9]. However, the optimal concurrent chemotherapy regimen remains uncertain [10]. Cisplatin-etoposide (EP) and weekly carboplatin-paclitaxel (PC) are the two most commonly used platinum-based regimens administered concurrently with thoracic radiotherapy. The EP regimen has traditionally been regarded as an effective concurrent regimen because full-dose chemotherapy can be safely delivered with radiotherapy while maintaining acceptable toxicity [11]. Conversely, the weekly PC regimen has gained widespread acceptance because of its convenient administration, favorable tolerability, and encouraging efficacy in patients with locally advanced unresectable NSCLC [12]. Despite their widespread clinical use, prospective studies directly comparing these two regimens remain limited [13]. The available evidence regarding their relative efficacy and toxicity is inconclusive [14].

Therefore, we conducted this prospective randomized study to compare the acute toxicity profiles and short-term tumor response of EP and weekly PC administered concurrently with definitive radiotherapy in patients with unresectable NSCLC. Six- and 12-month progression-free survival (PFS) and OS were examined as pre-specified exploratory secondary endpoints; given the modest sample size, this study was not powered to detect differences in survival, and all such comparisons should be interpreted as hypothesis-generating.

## Materials and methods

Study design and participants

This prospective randomized comparative study was conducted in the Department of Radiation Oncology, Sri Venkateswara Institute of Medical Sciences (SVIMS), Tirupati, India, between April 2021 and June 2022. Institutional Ethics Committee approval was obtained (ROC NO. AS/11/IEC/SVIMS/2017; Approval No. 1134), and all participants provided written informed consent. No formal sample-size calculation was performed; this is addressed as a limitation. This study was registered with the Clinical Trials Registry India (registration number: CTRI/2021/09/036500)

Patients aged 18-65 years with histologically confirmed unresectable stage IIB-IIIC NSCLC, an Eastern Cooperative Oncology Group (ECOG) performance status of 0-2, and adequate hematological, renal, and hepatic function were eligible. Stage IIB patients were included only when medically unfit for surgery. This determination was made by individual assessment by the treating radiation oncologist in consultation with a thoracic surgeon. ECOG performance status of 0-2 was applied strictly as an eligibility criterion at enrollment; consistent with the pre-specified exploratory analysis plan, performance status was not further stratified or statistically compared between treatment arms. Patients with distant metastases, malignant pleural effusion, previous lung cancer treatment, or clinically significant cardiovascular disease were excluded [15].

Treatment protocol

Patients underwent standard clinical, laboratory, histopathological, and radiological evaluation. Staging was performed using contrast-enhanced CT or whole-body fluorine-18 fluorodeoxyglucose positron emission tomography-computed tomography (18F-FDG PET-CT), with contrast-enhanced brain magnetic resonance imaging (MRI) when indicated. A computer-generated sequence randomized patients to weekly paclitaxel 45 mg/m² followed by carboplatin area under the curve (AUC) 2 on Days 1, 8, 15, 22, 29, and 36 (PC arm), or cisplatin 50 mg/m² on Days 1, 8, 29, and 36 with etoposide 50 mg/m² on Days 1-5 and 29-33 (EP arm).

All patients received concurrent definitive chemoradiotherapy according to standard institutional protocols. CT simulation was performed with 3-mm slices, and target volumes and organs at risk were delineated according to European Society for Radiotherapy and Oncology-Advisory Committee on Radiation Oncology Practice (ESTRO ACROP) recommendations [16]. Volumetric modulated arc therapy (VMAT) was delivered to a total dose of 60 Gy in 30 fractions over six weeks, with daily kilovoltage cone-beam computed tomography (kV-CBCT) image guidance; at least 95% of the planning target volume (PTV) was required to receive 95% of the prescribed dose [17]. Treatment plans were evaluated for adequate target-volume coverage and conformity while minimizing dose to organs at risk; plans were accepted when at least 95% of the PTV received at least 95% of the prescribed dose (PTV V95% ≥95%), significant dose hotspots were avoided, and the dose to the hottest 2 cm³ (D2cc) was preferably ≤120% of the prescribed dose. Predefined organ-at-risk dose constraints, based on established thoracic radiotherapy protocols and particularly Radiation Therapy Oncology Group (RTOG) 0617, were respected for conventional fractionation: mean lung dose ≤20 Gy and V20 ≤37%; mean esophageal dose ≤34 Gy; maximum spinal cord dose ≤50.5 Gy; and heart dose constrained to V60 <33% and V45 <66%. Identical treatment-planning and plan-evaluation principles were applied to patients in both treatment arms.

Outcomes and follow-up

The primary endpoint was acute treatment-related toxicity; secondary endpoints were three-month tumor response and PFS and OS at six and 12 months. Patients were assessed weekly during treatment and monthly for 12 months. Acute toxicities were graded using RTOG criteria [18], and tumor response was evaluated using Response Evaluation Criteria in Solid Tumors (RECIST) version 1.1 [19]. Acute toxicity was defined as toxicity occurring from the start of chemoradiotherapy through 90 days from the start of treatment, consistent with the RTOG acute toxicity convention. Radiation pneumonitis was diagnosed on the basis of new or increased radiographic opacity within the irradiated lung field on chest imaging, correlated with compatible respiratory symptoms, and graded per RTOG criteria.

PFS was calculated from the index date to progression or death, whichever occurred first; OS was calculated from the index date to death from any cause. Three patients lost before the three-month assessment were excluded, leaving 31 evaluable patients (PC, n=16; EP, n=15). Patients without documented progression or death were considered event-free.

Statistical analysis

Continuous variables were summarized as mean ± standard deviation (SD) and categorical variables as frequencies and percentages. Pearson’s chi-square (χ²) or Fisher-Freeman-Halton exact test was used as appropriate. PFS and OS were estimated separately at six and 12 months using the Kaplan-Meier method and compared using two-sided log-rank tests. Univariate Cox models estimated hazard ratios (HRs) and 95% confidence intervals (CIs) for EP relative to PC, with Breslow handling of ties. Restricted mean survival time (RMST) was calculated through each analysis horizon. All analyses were exploratory and two-sided, with p<0.05 considered statistically significant; no adjustment was made for multiple comparisons.

## Results

Patient characteristics

Following application of the predefined inclusion and exclusion criteria, 34 eligible patients were enrolled, comprising 18 patients in the concurrent PC arm and 16 patients in the concurrent EP arm.

All patients in both treatment arms completed the full planned chemotherapy regimen - all six weekly cycles of PC in the PC arm, and all planned cycles of EP in the EP arm; no chemotherapy cycle was omitted or permanently discontinued in either arm, and radiotherapy was delivered on schedule throughout, without interruption, in all patients. Hematological toxicity was managed primarily with supportive care, including blood transfusion for symptomatic or severe anemia and growth-factor support for neutropenia, rather than through permanent dose reduction or cycle omission. Where hematological recovery was incomplete on the scheduled treatment day, the affected chemotherapy cycle was briefly delayed until recovery, after which the full planned dose was administered; radiotherapy continued on the original schedule during any such brief chemotherapy delay. No patient required withholding of chemotherapy for renal toxicity. 

The baseline demographic and clinical characteristics according to treatment arm are summarized in Table 1. Overall, 29 patients (85.3%) were male, including 16/18 (88.9%) in the PC arm and 13/16 (81.3%) in the EP arm. The 51-60-year and 61-65-year age groups each included 15 patients (44.1%). Adenocarcinoma and squamous cell carcinoma were equally represented overall and within each treatment arm. Stage IIIA was the most common stage (14/34, 41.2%), followed by stage IIIB (12/34, 35.3%), stage IIIC (5/34, 14.7%), and stage IIB (3/34, 8.8%) (Table 1). All patients received external beam radiotherapy (EBRT) to a total dose of 60 Gy in 30 fractions concurrently with chemotherapy.

**Table 1:** Baseline patient demographics and clinical characteristics n: number of patients; EP: etoposide–cisplatin; PC: paclitaxel–carboplatin

Characteristic	Category	PC (n=18)	EP (n=16)	Total (n=34)
Age (years)	<40	0 (0.0%)	0 (0.0%)	0 (0.0%)
	41-50	1 (5.6%)	3 (18.8%)	4 (11.8%)
	51-60	8 (44.4%)	7 (43.8%)	15 (44.1%)
	61-65	9 (50.0%)	6 (37.5%)	15 (44.1%)
Sex	Male	16 (88.9%)	13 (81.3%)	29 (85.3%)
	Female	2 (11.1%)	3 (18.8%)	5 (14.7%)
Clinical stage	IIB	1 (5.6%)	2 (12.5%)	3 (8.8%)
	IIIA	8 (44.4%)	6 (37.5%)	14 (41.2%)
	IIIB	7 (38.9%)	5 (31.3%)	12 (35.3%)
	IIIC	2 (11.1%)	3 (18.8%)	5 (14.7%)
Histology	Adenocarcinoma	9 (50.0%)	8 (50.0%)	17 (50.0%)
	Squamous cell carcinoma	9 (50.0%)	8 (50.0%)	17 (50.0%)

Acute toxicity

Treatment-related hematological and non-hematological toxicities are summarized below.

Hematological Toxicity

The incidence of anemia differed significantly between the two treatment arms (p=0.006). Grade 1 and Grade 2 anemia occurred more frequently in the PC arm (38.9% and 55.6%, respectively), whereas Grade 3 and Grade 4 anemia were more common in the EP arm (6.3% and 43.8%, respectively). Only one patient (5.6%) in the PC arm developed Grade 3 anemia, while no patient developed Grade 4 anemia (Table 2).

**Table 2:** Anemia toxicity grading n: number of patients; PC: paclitaxel–carboplatin; EP: etoposide–cisplatin

Grade	PC (n=18)	EP (n=16)	Total (n=34)
Grade 1	7 (38.9%)	2 (12.5%)	9 (26.5%)
Grade 2	10 (55.6%)	6 (37.5%)	16 (47.1%)
Grade 3	1 (5.6%)	1 (6.3%)	2 (5.9%)
Grade 4	0 (0.0%)	7 (43.8%)	7 (20.6%)

Neutropenia occurred in both treatment groups without a statistically significant difference (p=0.110) (Table 3).

**Table 3:** Neutropenia toxicity grading n: number of patients; PC: paclitaxel–carboplatin; EP: etoposide–cisplatin

Grade	PC (n=18)	EP (n=16)	Total (n=34)
Grade 0	3 (16.7%)	0 (0.0%)	3 (8.8%)
Grade 1	10 (55.6%)	5 (31.3%)	15 (44.1%)
Grade 2	3 (16.7%)	5 (31.3%)	8 (23.5%)
Grade 3	2 (11.1%)	5 (31.3%)	7 (20.6%)
Grade 4	0 (0.0%)	1 (6.3%)	1 (2.9%)

No patient in either treatment arm developed Grade 2, Grade 3, or Grade 4 thrombocytopenia. Grade 1 thrombocytopenia was significantly more common in the EP arm than in the PC arm (81.3% vs. 38.9%, p=0.012) (Table 4).

**Table 4:** Thrombocytopenia toxicity grading n: number of patients; PC: paclitaxel–carboplatin; EP: etoposide–cisplatin

Grade	PC (n=18)	EP (n=16)	Total (n=34)
Grade 0	11 (61.1%)	3 (18.8%)	14 (41.2%)
Grade 1	7 (38.9%)	13 (81.3%)	20 (58.8%)
Grade 2–4	0 (0.0%)	0 (0.0%)	0 (0.0%)

Non-hematological Toxicity

Acute esophagitis differed significantly between the two treatment groups (p=0.006). Grade 1 esophagitis occurred more frequently in the PC arm (83.3%) than in the EP arm (31.3%). In contrast, Grade 2 and Grade 3 esophagitis were more common in the EP arm (56.3% and 12.5%, respectively). No patient in the PC arm developed Grade 3 esophagitis, and Grade 4 esophagitis was not observed in either treatment group (Table 5).

**Table 5:** Esophagitis toxicity grading n: number of patients; PC: paclitaxel–carboplatin; EP: etoposide–cisplatin

Grade	PC (n=18)	EP (n=16)	Total (n=34)
Grade 0	0 (0.0%)	0 (0.0%)	0 (0.0%)
Grade 1	15 (83.3%)	5 (31.3%)	20 (58.8%)
Grade 2	3 (16.7%)	9 (56.3%)	12 (35.3%)
Grade 3	0 (0.0%)	2 (12.5%)	2 (5.9%)
Grade 4	0 (0.0%)	0 (0.0%)	0 (0.0%)

Radiation pneumonitis was comparable between the two treatment arms (p=0.084). Grade 1 pneumonitis occurred more frequently in the EP arm (68.8%), whereas Grade 2 pneumonitis was more common in the PC arm (61.1%). One patient (5.6%) in the PC arm developed Grade 3 pneumonitis, whereas none occurred in the EP arm. No Grade 4 pneumonitis was observed in either treatment group (Table 6).

**Table 6:** Pneumonitis toxicity grading n: number of patients; PC: paclitaxel–carboplatin; EP: etoposide–cisplatin

Grade	PC (n=18)	EP (n=16)	Total (n=34)
Grade 0	0 (0.0%)	0 (0.0%)	0 (0.0%)
Grade 1	6 (33.3%)	11 (68.8%)	17 (50.0%)
Grade 2	11 (61.1%)	5 (31.3%)	16 (47.1%)
Grade 3	1 (5.6%)	0 (0.0%)	1 (2.9%)
Grade 4	0 (0.0%)	0 (0.0%)	0 (0.0%)

Tumor response at three months

Tumor response was assessed three months after completion of concurrent chemoradiotherapy using the RECIST version 1.1 (Table 7) [19].

**Table 7:** Tumor response grading n: number of patients; PC: paclitaxel–carboplatin; EP: etoposide–cisplatin

Response	PC (n=16)	EP (n=15)	Total (n=31)
Partial response	4 (25.0%)	11 (73.3%)	15 (48.4%)
Stable disease	7 (43.8%)	3 (20.0%)	10 (32.3%)
Progressive disease	5 (31.3%)	1 (6.7%)	6 (19.4%)

Of the 34 randomized patients, three did not attend the scheduled radiological response assessment and were therefore not evaluable for tumor response. Consequently, response evaluation was performed in the remaining 31 patients, including 16 patients in the PC arm and 15 patients in the EP arm.

Partial response was observed in 11 patients (73.3%) in the EP arm compared with four patients (25.0%) in the PC arm. Stable disease was observed in seven patients (43.8%) in the PC arm and three patients (20.0%) in the EP arm. Disease progression occurred in five patients (31.3%) in the PC arm and one patient (6.7%) in the EP arm. The overall distribution of tumor-response categories differed significantly between the two treatment arms (p=0.029).

PFS and OS

Survival analysis included 31 evaluable patients (PC, n=16; EP, n=15; Table 8). At six months, seven PFS events occurred with PC and five with EP. Six-month PFS was 56.3% (95% CI, 29.5%-76.2%) with PC and 66.7% (95% CI, 37.5%-84.6%) with EP. The difference was not statistically significant (log-rank χ²=0.457, p=0.499; Figure 1). The HR for progression or death with EP versus PC was 0.675 (95% CI, 0.214-2.131; Cox p=0.503). Six-month RMST was 169.7 days with PC and 174.3 days with EP.

**Table 8:** Six- and 12-Month PFS and OS analyses CI: confidence interval; EP: etoposide–cisplatin; HR: hazard ratio; OS: overall survival; PC: paclitaxel–carboplatin; PFS: progression-free survival; RMST: restricted mean survival time; KM: Kaplan–Meier

Analysis	Group	Events/N	KM estimate (95% CI)	HR, EP vs PC (95% CI)	Log-rank p; RMST (days)
6-month PFS	PC	7/16	56.3% (29.5%–76.2%)	0.675 (0.214–2.131)	0.499; 169.7
6-month PFS	EP	5/15	66.7% (37.5%–84.6%)		174.3
12-month PFS	PC	12/16	25.0% (7.8%–47.2%)	0.574 (0.239–1.375)	0.206; 235.8
12-month PFS	EP	9/15	40.0% (16.5%–62.8%)		285.5
6-month OS	PC	3/16	81.3% (52.5%–93.5%)	0.333 (0.035–3.201)	0.317; 179.8
6-month OS	EP	1/15	93.3% (61.3%–99.0%)		182.2
12-month OS	PC	7/16	56.3% (29.5–76.2%)	0.380 (0.098–1.472)	0.144; 313.7
12-month OS	EP	3/15	80.0% (50.0–93.1%)		347.7

**Figure 1:**
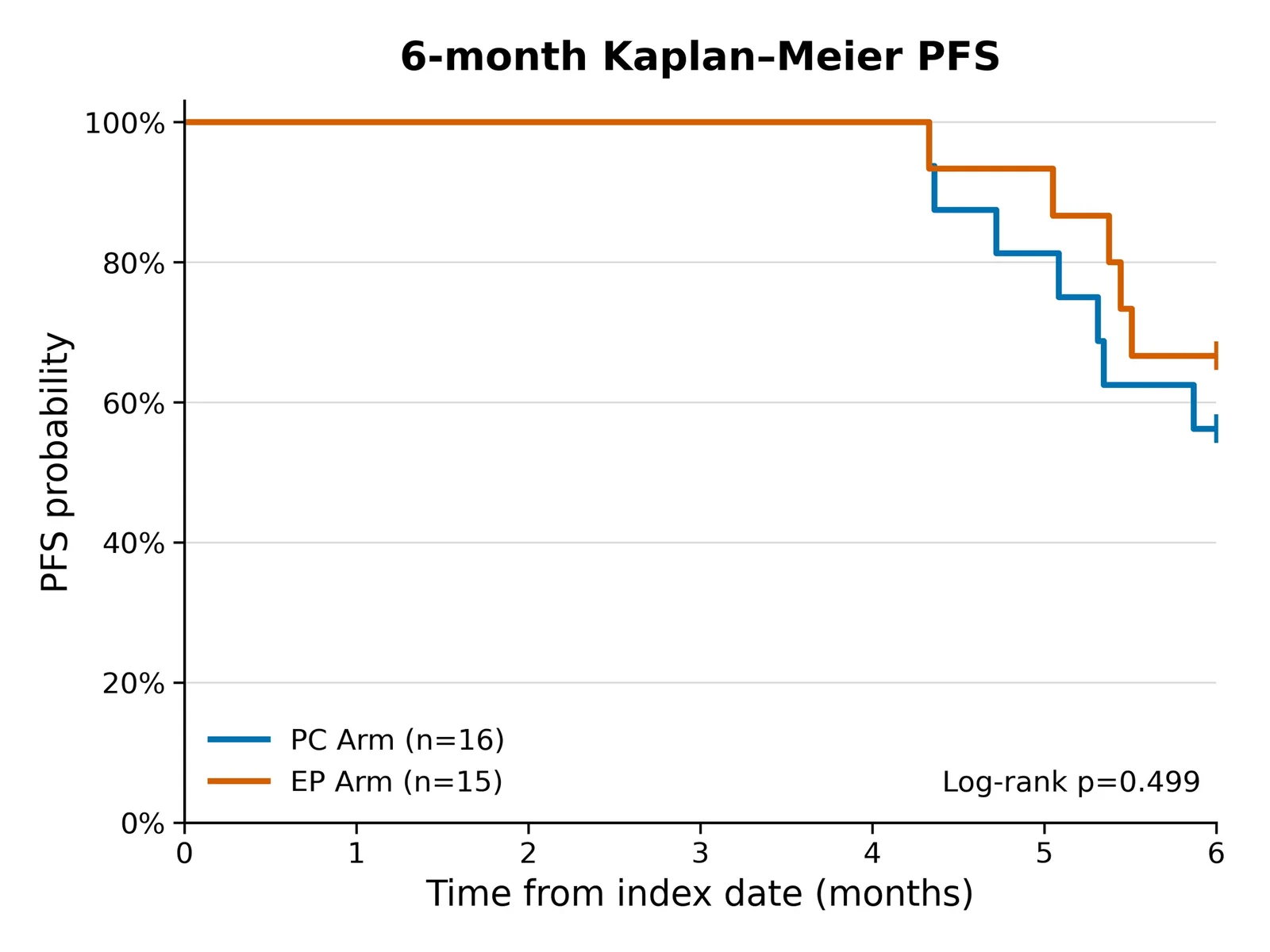
Six-month Kaplan–Meier curve for PFS PFS: progression-free survival; PC: paclitaxel–carboplatin; EP: etoposide–cisplatin

At 12 months, 12 PFS events occurred with PC and nine with EP. Median PFS was 7.36 months (95% CI, 5.09-8.97 months) with PC and 11.20 months (95% CI, 5.39 months to not estimable) with EP. Twelve-month PFS was 25.0% (95% CI, 7.8%-47.2%) and 40.0% (95% CI, 16.5%-62.8%), respectively. The difference was not statistically significant (log-rank χ²=1.597, p=0.206; Figure 2). The HR was 0.574 (95% CI, 0.239-1.375; Cox p=0.213), and 12-month RMST was 235.8 days with PC and 285.5 days with EP.

**Figure 2:**
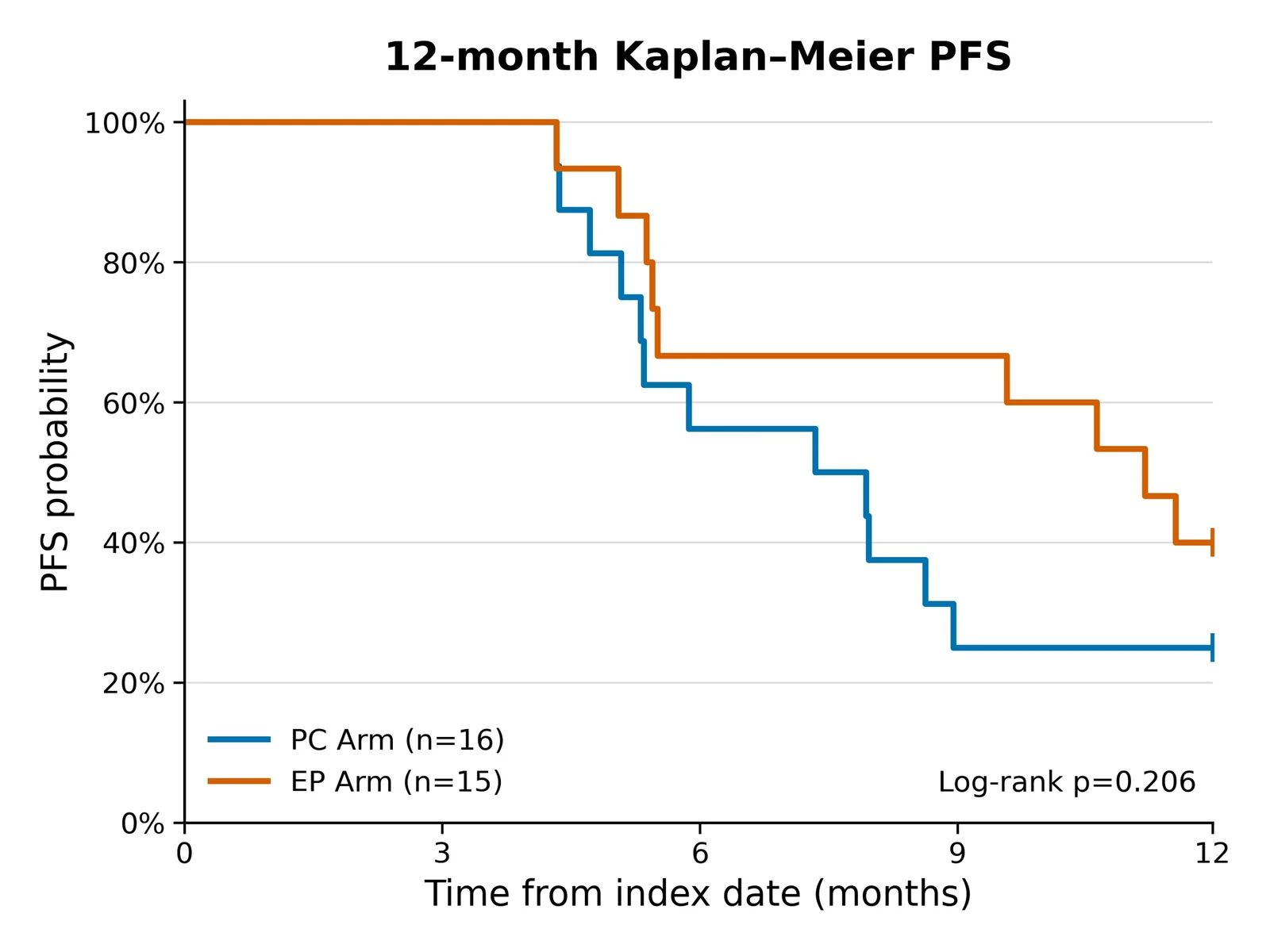
Twelve-month Kaplan–Meier curve for PFS PFS: progression-free survival; PC: paclitaxel–carboplatin; EP: etoposide–cisplatin

For six-month OS, three deaths occurred with PC and one with EP. Six-month OS was 81.3% (95% CI, 52.5%-93.5%) with PC and 93.3% (95% CI, 61.3%-99.0%) with EP. The difference was not statistically significant (log-rank χ²=1.003, p=0.317; Figure 3). The HR for death with EP versus PC was 0.333 (95% CI, 0.035-3.201; Cox p=0.341). Six-month RMST was 179.8 days with PC and 182.2 days with EP.

**Figure 3:**
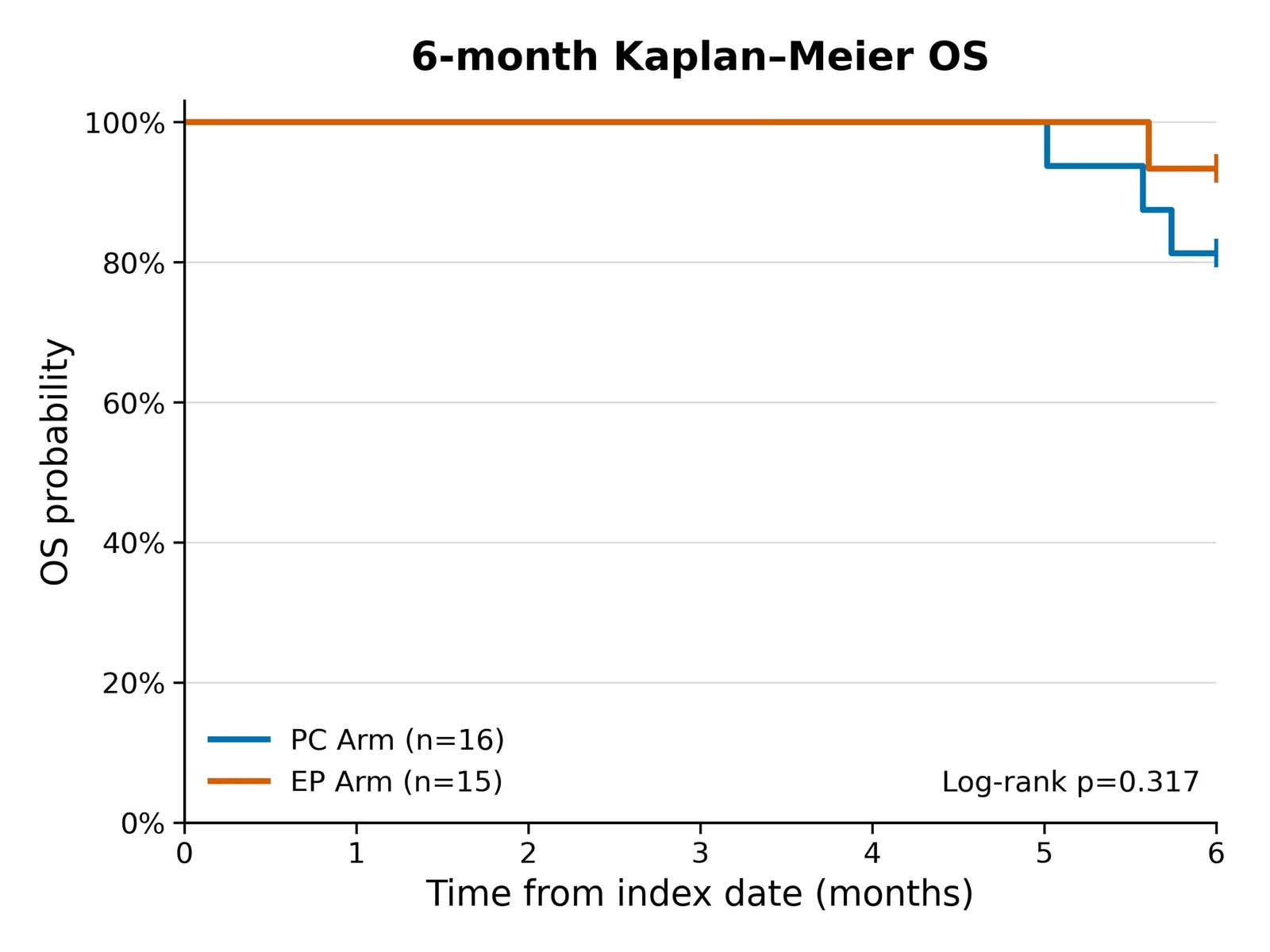
Six-month Kaplan–Meier curve for OS OS: overall survival; PC: paclitaxel–carboplatin; EP: etoposide–cisplatin

For 12-month OS, seven deaths occurred with PC and three with EP. Median OS was not reached in either arm. Twelve-month OS was 56.3% (95% CI, 29.5%-76.2%) with PC and 80.0% (95% CI, 50.0%-93.1%) with EP. The difference was not statistically significant (log-rank χ²=2.133, p=0.144; Figure 4). The HR was 0.380 (95% CI, 0.098-1.472; Cox p=0.161), and 12-month RMST was 313.7 days with PC and 347.7 days with EP.

**Figure 4:**
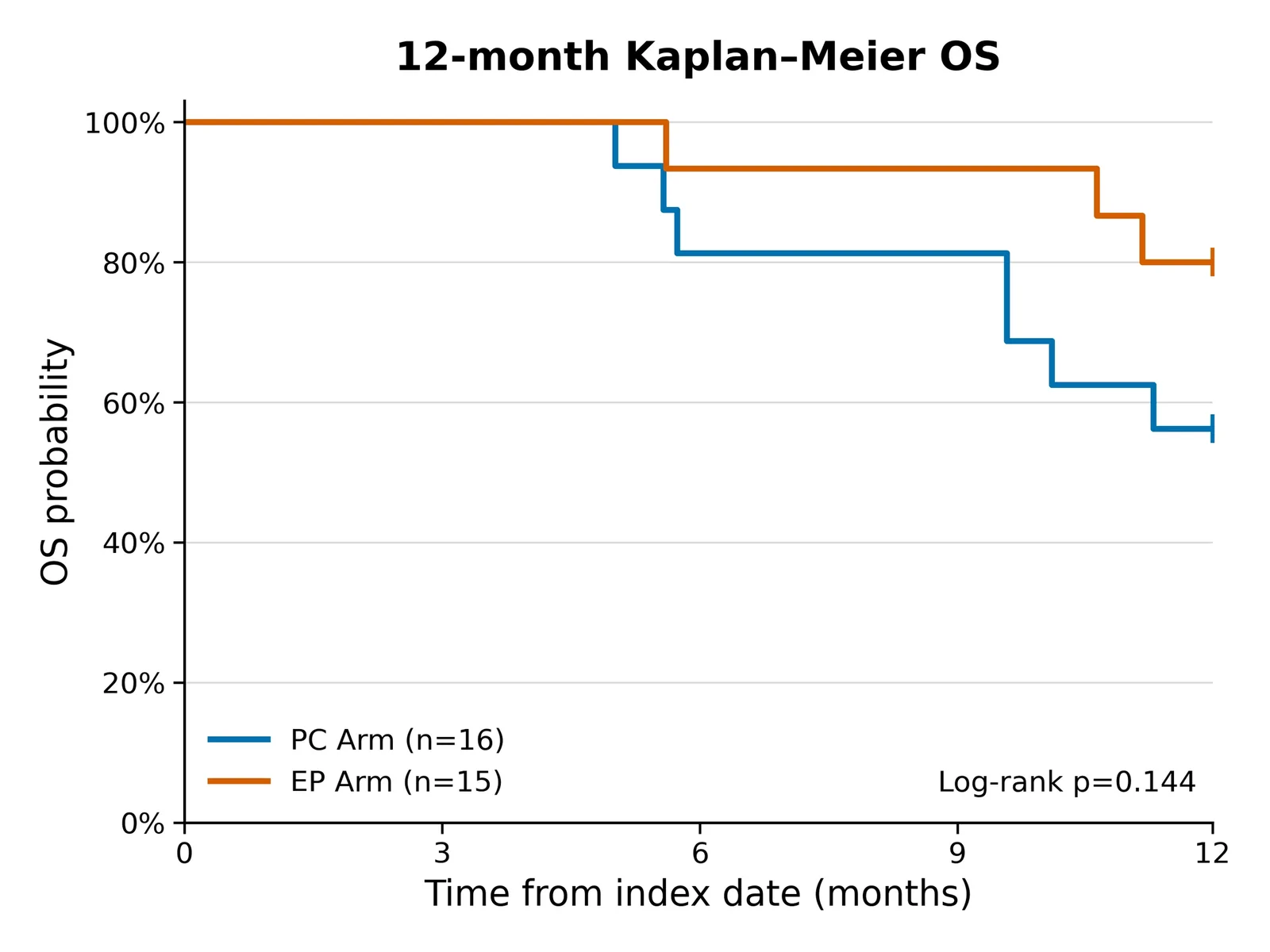
Twelve-month Kaplan–Meier curve for OS OS: overall survival; PC: paclitaxel–carboplatin; EP: etoposide–cisplatin

## Discussion

This prospective randomized study compared concurrent EP with weekly PC during definitive radiotherapy for unresectable NSCLC. EP produced a more favorable three-month tumor-response distribution and numerically better one-year PFS and OS, but at the cost of greater hematological toxicity and esophagitis.

Toxicity

The study findings are consistent with comparative studies and the systematic review by Steuer et al., which reported greater hematological toxicity with EP [14]. Pneumonitis was numerically more frequent with PC but was not significantly different between groups, a pattern also reported in previous comparative studies [20]. Severe anemia and Grade 1 thrombocytopenia were significantly more frequent with EP, while neutropenia was also numerically higher [21]. Acute esophagitis was significantly more severe with EP, consistent with the randomized phase III trial by Liang et al. [22].

The higher response observed with EP may be related to the delivery of a full-dose cisplatin-based doublet during radiotherapy. Cisplatin provides potent radiosensitization through inhibition of DNA repair, while etoposide increases DNA damage by inhibiting topoisomerase II. This is a plausible, hypothesis-generating mechanistic explanation rather than one directly tested by the present study; the combination may enhance locoregional tumor killing but also exposes proliferating bone-marrow and esophageal tissues to greater combined cytotoxic injury. Although treatment delivery was complete in all patients, we cannot exclude that unmeasured differences in radiotherapy dosimetry or post-chemoradiotherapy systemic treatment between arms also contributed to the observed toxicity and efficacy differences. Conversely, weekly low-dose PC provides continuous radiosensitization with lower individual chemotherapy doses and is generally easier to administer, which may partly explain its more favorable hematological and esophageal toxicity profile [14].

Tumor response

Among the 31 evaluable patients, the partial-response proportion was 73.3% with EP and 25.0% with PC, and the overall response distribution differed significantly (p=0.029). This direction is compatible with randomized evidence suggesting improved disease control with EP [22]. However, the systematic review by Steuer et al. found no significant difference in pooled response rates, indicating that the magnitude of the present difference may reflect the small sample size, patient selection, or treatment-related factors [14].

The marked difference in partial-response proportions should nevertheless be interpreted cautiously. Early radiological response reflects short-term tumor shrinkage and may indicate improved locoregional control, but it does not necessarily translate into a proportional survival benefit. Subsequent distant failure, treatment-related morbidity, competing comorbidities, and access to salvage or maintenance treatment can modify long-term outcomes. The small number of evaluable patients also makes the response estimate vulnerable to chance imbalance between treatment groups. In addition, three randomized patients were excluded from the response and survival analyses because they were lost to follow-up before the three-month assessment; if loss to follow-up was related to underlying disease severity or treatment tolerance, this exclusion could introduce attrition bias favoring either arm, and the response and survival estimates reported here should be interpreted with this possibility in mind.

PFS and OS

The direction of effect favored EP at both analysis horizons. Six-month PFS was 66.7% with EP and 56.3% with PC (HR, 0.675; log-rank p=0.499), while 12-month PFS was 40.0% and 25.0%, respectively (HR, 0.574; p=0.206). Similarly, six-month OS was 93.3% with EP and 81.3% with PC (HR, 0.333; p=0.317), and 12-month OS was 80.0% and 56.3%, respectively (HR, 0.380; p=0.144). Median PFS was 11.20 months with EP and 7.36 months with PC, whereas median OS was not reached. The PFS estimates are directionally consistent with the systematic review reporting median PFS of approximately 12 months with EP and 9.3 months with PC without a significant difference [14].

The consistently favorable numerical estimates with EP across six- and 12-month PFS and OS support a possible efficacy signal, but none of the separate comparisons was statistically significant. Given the small number of events and the correspondingly wide 95% CIs around every HR reported, each of which spans both clinically meaningful benefit and clinically meaningful harm, these numerically favorable estimates should not be interpreted as evidence of superior survival efficacy with EP. They are best regarded as an exploratory signal warranting confirmation in an adequately powered trial, not as a basis for preferring one regimen over the other on survival grounds. Wang et al. reported improved long-term survival with EP [20], and Liang et al. reported a median OS of 23.3 months with EP versus 20.7 months with PC, with three-year OS favoring EP [22]. Conversely, the Veterans Health Administration analysis found no adjusted survival advantage with EP and reported greater treatment-related morbidity [23]. The present findings are therefore compatible with the mixed comparative literature and should be regarded as hypothesis-generating rather than evidence of regimen superiority.

The absence of statistical significance despite numerically favorable survival with EP is likely related to limited statistical power. The six-month analyses included only 12 PFS events and four deaths, producing particularly wide HR CIs; by 12 months, 21 PFS events and 10 deaths had occurred, but precision remained limited. Separate censoring at day 183 and day 365 appropriately answers horizon-specific questions but does not create independent patient cohorts, and the four comparisons increase the possibility of chance findings. Follow-up restricted to one year also prevents assessment of later curve separation or convergence and leaves median OS unreached. Moreover, classifying patients without documented progression or death as event-free through the relevant horizon may overestimate survival, while excluding three patients lost before three months may introduce attrition bias. Accordingly, the HRs, RMST differences, and Kaplan-Meier estimates should be interpreted as exploratory estimates with substantial uncertainty.

Overall, EP may provide greater tumor control in selected fit patients but produces more hematological and esophageal toxicity. Weekly PC offers greater convenience and tolerability and may be a reasonable option to consider in patients less likely to tolerate full-dose platinum-based chemotherapy, such as those with reduced performance status or significant comorbidity, although this study was not designed or powered to support a specific treatment-selection recommendation. Regimen selection should therefore consider performance status, organ function, expected tolerance, and patient preference rather than assuming the superiority of either regimen.

Limitations

The present study has several limitations. Its single-center, exploratory design and small sample size, without a formal a priori sample-size calculation, limit statistical power and generalizability. Consequently, the PFS and OS comparisons in particular were underpowered, and the numerically favorable estimates for EP at both six and 12 months should not be construed as evidence of a survival advantage. Three randomized patients were lost to follow-up before the three-month assessment and were excluded from the evaluable response and survival analyses, which may introduce attrition bias. The short one-year follow-up precludes reliable assessment of long-term survival, and no adjustment was made for multiple comparisons. The study was not designed to assess late toxicity, patterns of failure, subsequent systemic therapy, or contemporary consolidation immunotherapy. Several potentially relevant baseline prognostic factors such as smoking status, comorbidity burden, baseline hepatic function, tumor size/burden, and continuous baseline hematological and renal parameters were not systematically recorded in a form permitting by-arm comparison, and their distribution between arms is therefore unknown. The absence of these baseline variables is a reporting limitation affecting our ability to fully characterize the study population, rather than a flaw in the randomized comparison of the prespecified toxicity and response endpoints, which remain valid as measured. The study was open-label; blinding of a chemoradiotherapy regimen is logistically infeasible, but the lack of blinding may have introduced assessment bias, particularly in grading of subjective toxicities such as esophagitis, where assessors were aware of treatment assignment. Patient-reported outcomes and formal quality-of-life assessment were not collected; given that survival did not differ significantly between arms while toxicity did, such measures would be an important input into regimen selection, and their absence is a notable limitation of this study. Nevertheless, these findings provide useful preliminary evidence and support further evaluation in larger, adequately powered multicenter studies with longer follow-up.

## Conclusions

Despite these limitations, this prospective randomized study provides comparative evidence regarding two commonly used CCRT regimens for unresectable locally advanced NSCLC. EP produced a more favorable short-term tumor-response distribution and numerically higher one-year PFS and OS estimates, which were not statistically significant and should be interpreted as an exploratory signal rather than evidence of a survival benefit, but the survival differences were not statistically significant. These potential efficacy benefits were accompanied by greater hematological toxicity and acute esophagitis, whereas weekly PC demonstrated better tolerability. This study was underpowered to detect differences in survival outcomes, and no definitive conclusion regarding the superiority of either regimen can be drawn. Larger multicenter randomized studies with complete long-term follow-up are required to determine whether either regimen provides a clinically meaningful survival advantage.
